# SENP3 affects the expression of PYCR1 to promote bladder cancer proliferation and EMT transformation by deSUMOylation of STAT3

**DOI:** 10.18632/aging.204333

**Published:** 2022-10-11

**Authors:** Zhuo Li, Jian Liu, Huifeng Fu, Yuanwei Li, Qiang Liu, Wei Song, Mingqiang Zeng

**Affiliations:** 1 Department of Urology, Hunan Provincial People’s Hospital, The First Affiliated Hospital of Hunan Normal University, Changsha City, Hunan Province 410005, China

**Keywords:** bladder cancer, signal transducer and activator of transcription 3, sentrin/SUMO-specific protease 3, SUMOylation, pyrroline-5-carboxylate reductase 1, epithelial mesenchymal transformation

## Abstract

Abnormal activation of signal transducer and activator of transcription 3 (STAT3) has been found in various types of human cancers, including bladder cancer (BC). In our study, we examined the regulation of STAT3 post-translational modifications (PTMs) and found that SENP3 is high in bladder cancer. Sentrin/SUMO-specific protease3 (SENP3) and STAT3 were highly expressed in BC tissues when compared with tissue adjacent to carcinoma. SENP3 induced STAT3 protein level and p-STAT3 translocating into nuclear through deSUMOylation of STAT3. Further, nuclear STAT3, as a transcriptional activity factor, promoted pyrroline-5-carboxylate reductase 1 PYCR1 gene and protein level by interacting with the promoter of (PYCR1). Next, we found that knockdown of PYCR1 inhibited Epithelial to mesenchymal transition of bladder cancer, and simultaneously mitigated the carcinogenic effects of STAT3. *In vitro*, STAT3 knockdown in bladder cancer cells inhibited cell proliferation, migration, and invasion. In contrast, SENP3 overexpression reversed these effects. In all, results lend novel insights into the regulation of STAT3, which has key roles in bladder cancer progression.

## INTRODUCTION

Bladder cancer (BC) is the 7th common cancers in men and the 17th in women worldwide [1, 2]. To date, there are about 550,000 people diagnosed with bladder cancer, accounting for about 3% of all newly diagnosed cancers [3]. The incidence of bladder cancer normally increases with age, but some unhealthy lifestyle, such as smoking also increased the incidence [4]. Although much effort has been paid to the therapy, the five-year relative survival rates of advanced bladder cancer have little improvement in recent years [5–7].

As a transcription factor, signal transducer and activator of transcription 3 (STAT3) exerts important effects on cellular growth, survival and inflammatory response [8, 9]. For instance, STAT3 transcriptionally regulates CCR7 to promotes cancer, while miR-4500 inhibits STAT3 expression by targeting the STAT3 3′-3UTR region and thus suppressed cancer development [10]. Additionally, STAT3 induces the resistance of prostate cancer to chemoradiation and small-molecule inhibitors, reflecting the roles in cancer development [11]. The transcriptional activity of STAT3 is regulated by post-translational modifications (PTMs) in the protein [12]. For example, the phosphorylation of tyrosine 705 induces STAT3 translocation to the nucleus, where STAT3 functions to the transcription of target genes [13, 14]. Various types of cancers show hyperactivation of STAT3 by tyrosine 705 phosphorylation, which greatly contributes to tumor development. It has been reported that phospho-STAT3 (p-STAT3) induces methylation of estrogen receptor 1 (ESR1) promoter in bladder cancer [15], and RacGAP1 induces phosphorylation of STAT3 [16], which increases the expression of p-STAT3 and promotes its translocation into the nucleus to play a cancer-promoting role. Knocking-down of sentrin/SUMO-specific protease3 (SENP3) greatly hinders STAT3 phosphorylation induced by tobacco [17]. Dysregulation has been identified in solid tumor and STAT3 activation is a marker for poor outcome [18]. The aim was to explore potential therapeutic strategies by targeting this pathway in BC.

SUMO modification, a reversible post-translational modification, is involved in multiple cellular processes. To date, SUMO1, SUMO2, and SUMO3 have found to induce protein SUMOylation [19, 20]. SENP family proteins, including SENP1, 2, 3, 5, 6, 7, and 8, conversely induced the de-SUMOylation [19, 21]. Dynamics of SUMO and de-SUMO modifications to great extent influence protein activity, protein-protein interaction, localization, thereby impacting cellular behaviors [22, 23]. SENP3 is widely involved in a variety of diseases, including tumors, and has not been reported in bladder cancer [24]. SENP3 positively regulates the activation of STAT3 by promoting deSUMOylation of STAT3 in head and neck cancer after the simulation of tobacco [16]. Here, we demonstrated deSUMOylation STAT3 by SENP3, which was activated to accumulated nuclear. We aimed to further investigate the association between SUMOylation and bladder cancer proliferation and epithelial mesenchymal transformation (EMT) in this study.

STAT3 in the nucleus upregulated the expression of oncogene pyrroline-5-carboxylate reductase 1 (PYCR1), which promoted bladder cancer proliferation and EMT. PYCR1 is an enzyme responsible for cell metabolism and is upregulated in cancer [25]. PYCR1 functions as an oncogene in bladder cancer. In addition, PYCR1 is up-expressed in numerous malignancies, including bladder cancer, breast cancer, renal cell cancer and lung cancer [25–28].

The aim of the present study was to explore the effect and mechanism of STAT3 and its activation in bladder cancer. We sought to clarify how SENP3 affects PYCR1 and STAT3 to regulate the development of bladder cancer.

## RESULTS

### SENP3 protein level correlates with protein expression of STAT3 and p-STAT3 in bladder cancer

**Figure 1:**
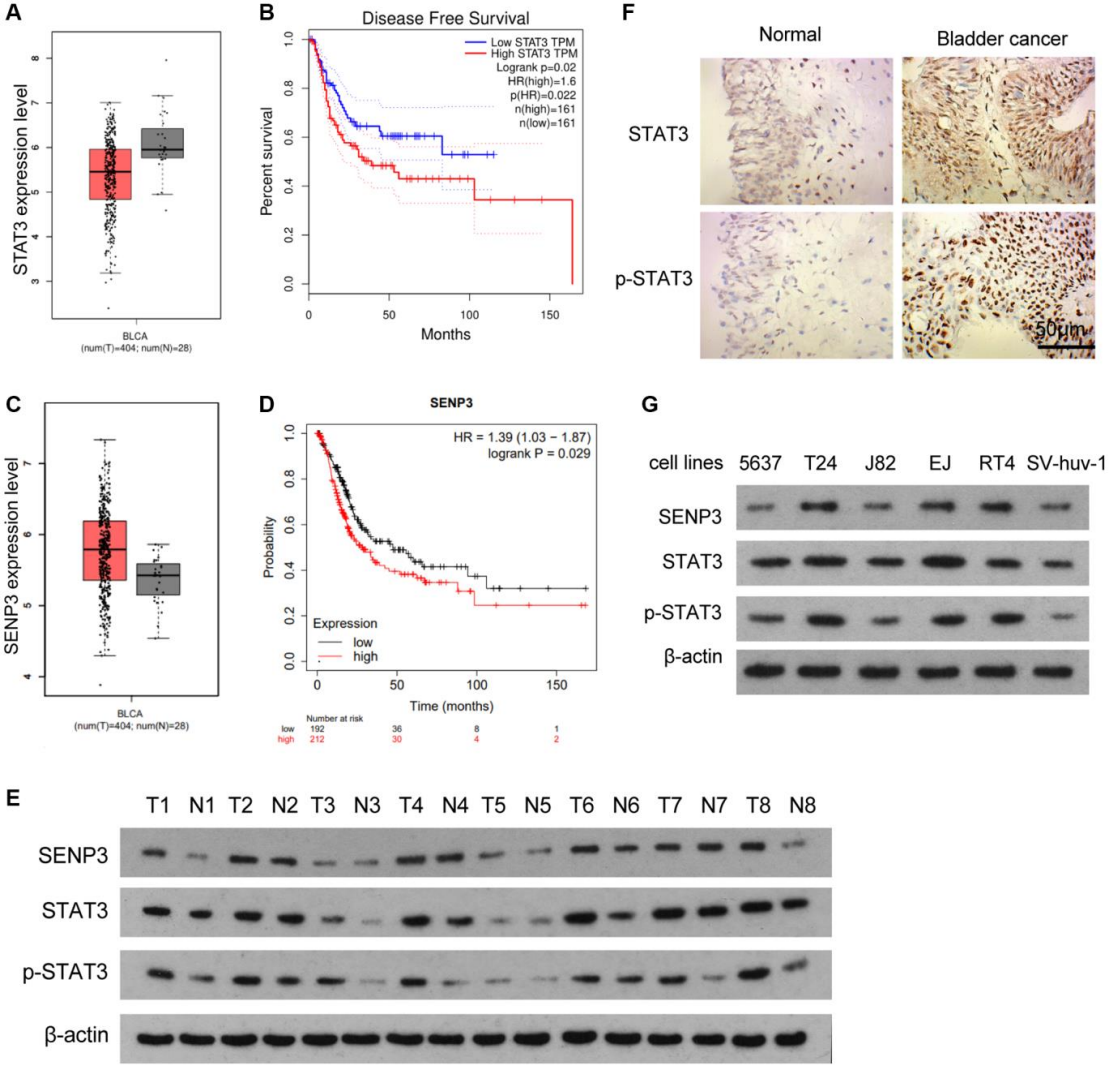
**SENP3 protein level correlates with protein expression of STAT3 and p-STAT3 in bladder cancer.** (**A**) Differences of STAT3 between bladder cancer tissues and adjacent normal tissues, determined by bioinformatics analysis. (**B**) Disease free survival with low and high STAT3 TPM. (**C**) SENP3 level between bladder cancer tissues and adjacent normal tissues, determined by bioinformatics analysis. (**D**) The probability of cancer progression plotted over a period of 150 months for patients with high or low SENP3 expressions. (**E**) SENP3, STAT3 and p-STAT3 protein levels in bladder cancer tissues and adjacent normal tissues, as measured by western blot. (**F**) Expression of STAT3 and p-STAT3 in bladder cancer tissues and adjacent normal tissues by immunohistochemistry [scale bar, 50 μm]. (**G**) SENP3, STAT3 and p-STAT3 protein levels in 5637, T24, J82, EJ, RT4 and SV-huv-1 cells.

The GEPIA database of bladder cancer showed that STAT3 expression in bladder cancer tissues was lower than that in adjacent normal tissues (Figure 1A). However, survival curve analysis showed that the prognosis of patients with high STAT3 expression was poor (Figure 1B). The contrast between gene expression level and gene function suggested that STAT3 might have post-translational protein modification. STAT3 has been reported to be SUMOylation in tumors [16], and SENP3 expression in bladder cancer tissue was higher than that in adjacent or normal tissue (Figure 1C). Survival curve analysis showed that the prognosis of patients with high SENP3 expression was poor (Figure 1D). When we assessed the effect of SENP3 expression in bladder cancer patients (400 samples), the probability of cancer progression was found to be statistically significant with low SENP3 expression as compared to that of high SENP3 expression. Meanwhile, SENP3 promoted the phosphorylation of STAT3 to address the relationship between SENP3 and STAT3 in bladder cancer, SENP3 and STAT3 proteins were detected in 8 cases of bladder cancer and para-cancerous tissues. The results showed that the expression levels of SENP3, STAT3 and p-STAT3 in bladder cancer tissues were higher than para-cancerous normal tissues (Figure 1E). At the same time, we detected STAT3 and p-STAT3 in bladder cancer and para-cancerous normal tissues by IHC assay. The protein level of STAT3 and p-STAT3 in bladder cancer tissues was higher than that in normal tissues, especially the strong staining signal of p-STAT3 in the nucleus (Figure 1F). In order to further detect the regulation of STAT3 and p-STAT3 by SENP3, two cell lines (T24 cells and EJ cells) with higher STAT3 protein expression level were selected for subsequent experiments (Figure 1G). Taken together, SENP3 protein level is up-regulated in bladder cancer, which is correlates with protein expression of STAT3 and p-STAT3.

### SENP3 promotes proliferation and deSUMOylation of STAT3

**Figure 2:**
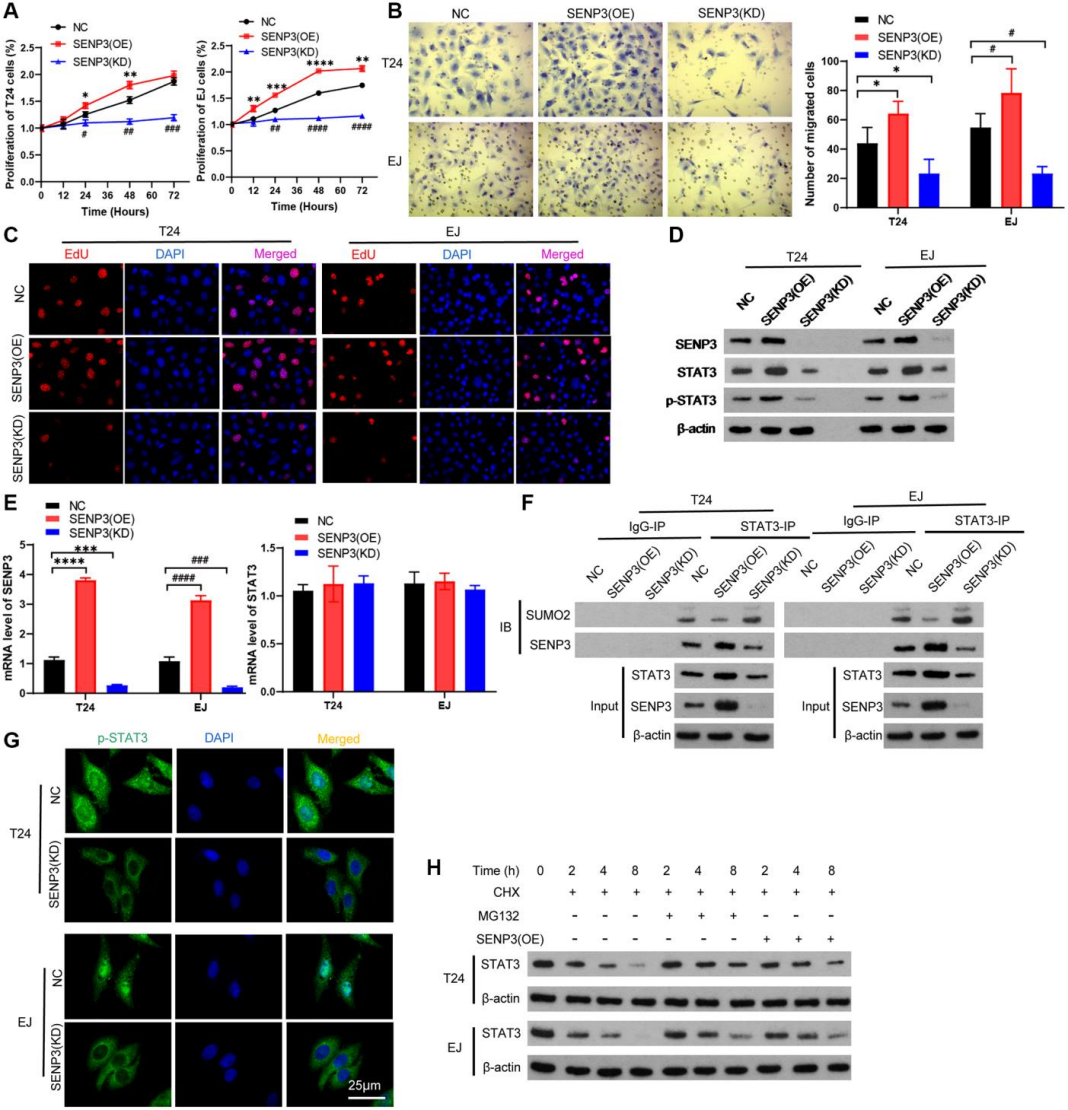
**SENP3 promotes proliferation and deSUMOylation of STAT3.** T24 and EJ cells were transfected plasmids with overexpression of SENP3 [STAT3(OE)] and knock-down of SENP3 [STAT3(KD)]. (**A**) Cell proliferation assay *in vitro* in NC, SENP3(OE), SENP3(KD) T24 and EJ cells. [mean ± S.D. (error bars), *n* = 3. ^**^p ≤ 0.01; ^***^p ≤ 0.001; ^****^p ≤ 0.0001, compared with NC group; ^##^*p* ≤ 0.01; ^####^*p* ≤ 0.0001, compared with NC group, two-way analysis of variance]. (**B**) Left: Cell invasion determined by trans well staining in SENP3 overexpression or knockdown T24 and EJ cells. [scale bar, 25 μm]. right: quantitative analysis of immunohistochemistry for positive trans well staining. [mean ± S.D. (error bars), *n* = 3. ^*^p ≤ 0.05, compared in T24 cells; ^#^*p* ≤ 0.05, compared in EJ cells, two-way analysis of variance] (**C**) EDU positive cells in NC, SENP3(OE), SENP3(KD) T24 and EJ cells determined by Confocal immunofluorescence. [scale bar, 50 μm]. (**D**) SENP3, STAT3 and p-STAT3 protein levels in NC, SENP3(OE), SENP3(KD) T24 and EJ cells, as measured by western blot. (**E**) The mRNA level of SENP3 and STAT3 measured by qPCR. (**F**) Co-immunoprecipitation (co-IP) of endogenous SENP3 with STAT3 and its SUMO2. (**G**) Abundance of p-STAT3 protein in in NC, SENP3(OE) T24 and EJ cells. (**H**) T24 cells were transfected with the indicated constructs and treated for the indicated times with CHX and MG132, whole cells were collected and STAT3 protein level was determined by western blot. All experiments were performed in triplicates.

To further explore the mechanism of STAT3 regulated by SENP3, we constructed SENP3 stably knockdown and overexpression T24 cells and EJ cells. Both CCK8 assay and EDU immunofluorescence staining showed that overexpression of SENP3 promoted cell proliferation (Figure 2A and Figure 2C). Trans well staining indicated that SENP3 promoted cell invasion (Figure 2B). To explore regulated effect of STAT3 by overexpression and knockdown of SENP3 in T24 cells and EJ cells, we detected gene and protein levels of STAT3 (Figure 2D). SENP3 up-regulated the STAT3 protein level, not affect the gene expression of STAT3 (Figure 2E and Figure 2F). Meanwhile, SENP3 promotes the stability of STAT3 protein by regulating STAT3 de-sumo modification [16], and the increase of intracellular STAT3 up-regulates p-STAT3 protein level (Figure 2D and 2F). To test whether SENP3 de-conjugates the SUMO modification of STAT3 and how to regulate it, immunoprecipitation assay suggested that SENP3 physically associates with STAT3 (Figure 2G). When SENP3 binding to STAT3, STAT3-SUMOylation level significantly decreased. To investigate the stability of desumoylated-STAT3 regulated by SENP3, treating with Cycloheximide (CHX) and MG132, overexpression of SENP3 delayed the degradation of STAT3, while knock-down of SENP3 accelerated the degradation of STAT3 (Figure 2H). SENP3 improved the protein stability of STAT3. These results indicate that SENP3 promotes proliferation and deSUMOylates to stabilize STAT3 protein level.

### STAT3 mitigates the cancer-promoting effect of SENP3

**Figure 3:**
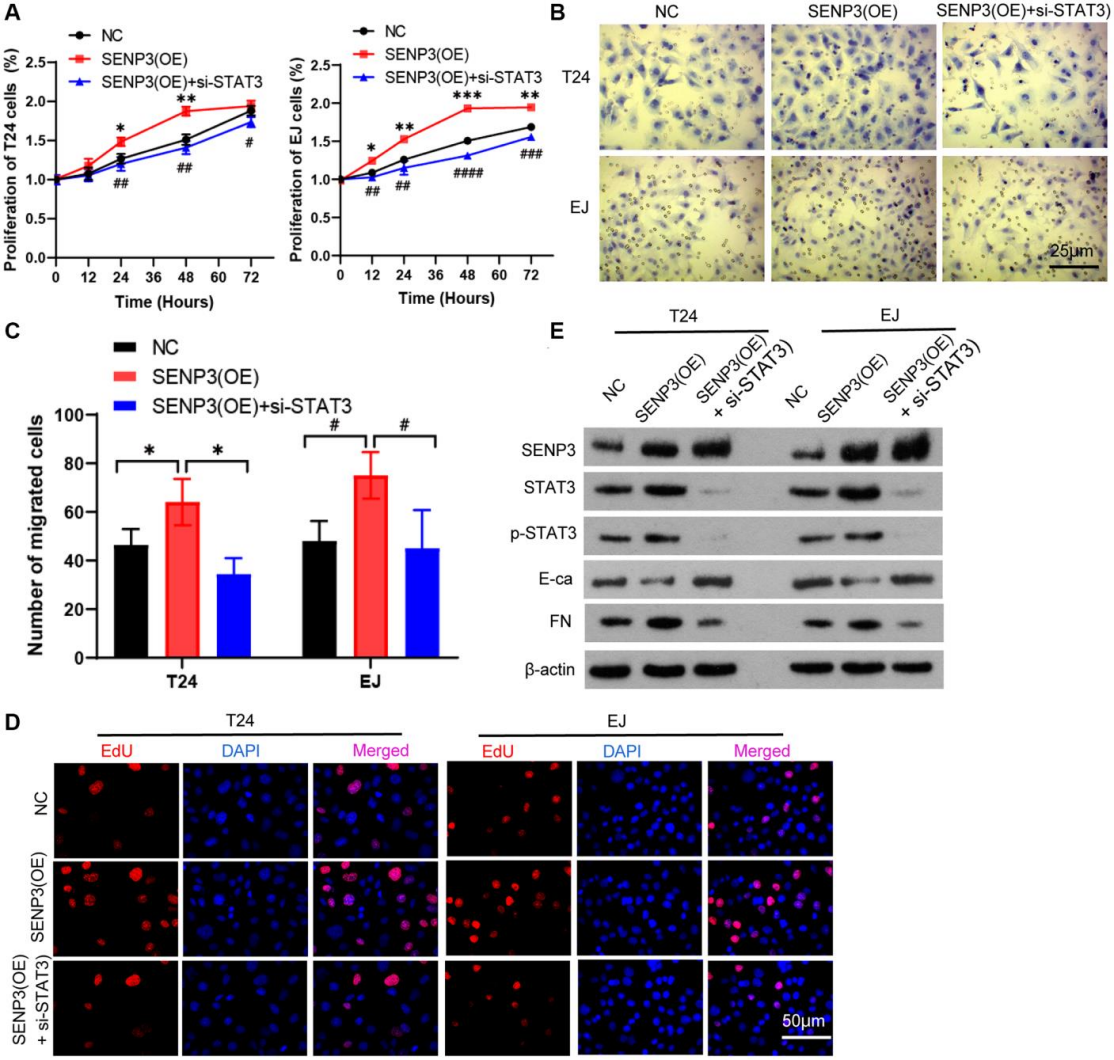
**STAT3 mitigates the cancer-promoting effect of SENP3.** (**A**) Overexpression of SENP3 [STAT3(OE)] T24 and EJ cells were transfected with STAT3 siRNAs [SENP3(OE) + si-STAT3]. Cell proliferation were determined by CCK8 essay in NC, SENP3(OE), SENP3(OE) + si-STAT3 T24 and EJ cells. [mean ± S.D. (error bars), *n* = 3. ^*^p ≤ 0.05; ^**^p ≤ 0.01; ^***^p ≤ 0.001, compared with NC group; ^#^*p* ≤ 0.05; ^##^*p* ≤ 0.01; ^###^*p* ≤ 0.001; ^####^*p* ≤ 0.0001, compared with SENP(OE) group, two-way analysis of variance]. (**B**) Cell invasion determined by trans well staining in NC, SENP3(OE), SENP3(OE) + si-STAT3 T24 and EJ cells. (**C**) Analysis of cell migration. [mean ± S.D. (error bars), *n* = 3. ^*^p ≤ 0.05, compared in T24 cells; ^#^*p* ≤ 0.05, compared in EJ cells, two-way analysis of variance]. (**D**) EDU positive cells in NC, SENP3(OE), SENP3(OE) + si-STAT3 T24 and EJ cells determined by Confocal immunofluorescence. (**E**) Abundance of the indicated protein was analyzed by Western blotting. All experiments were performed in triplicates.

SENP3 is a cancer-promoting protein, which up-regulates protein level of STAT3. We test whether silencing STAT3 reverses the cancer-promoting effect of SENP3. When knocking down STAT3 in overexpression of SENP3 cells, cell proliferation and EDU positive staining were up-regulated, which closing to or lower than control group (Figure 3A). Trans well staining indicated that Knocking-down of SENP3 clearly inhibited cell invasion comparing with overexpression of SENP3 (Figure 3B and 3C), knock-down of STAT3 significantly reduced p-STAT3 protein level. Overexpression of SENP3 promoted EMT by reducing E-Ca and increasing FN protein levels. When knocking down STAT3, the changes of E-Ca and FN were reversed (Figure 3D and 3E). These data suggest that STAT3 mitigates the cancer-promoting effect of overexpression of SENP3.

### STAT3 promotes gene and protein levels of PYCR1 by binding to promoter of PYCR1

**Figure 4:**
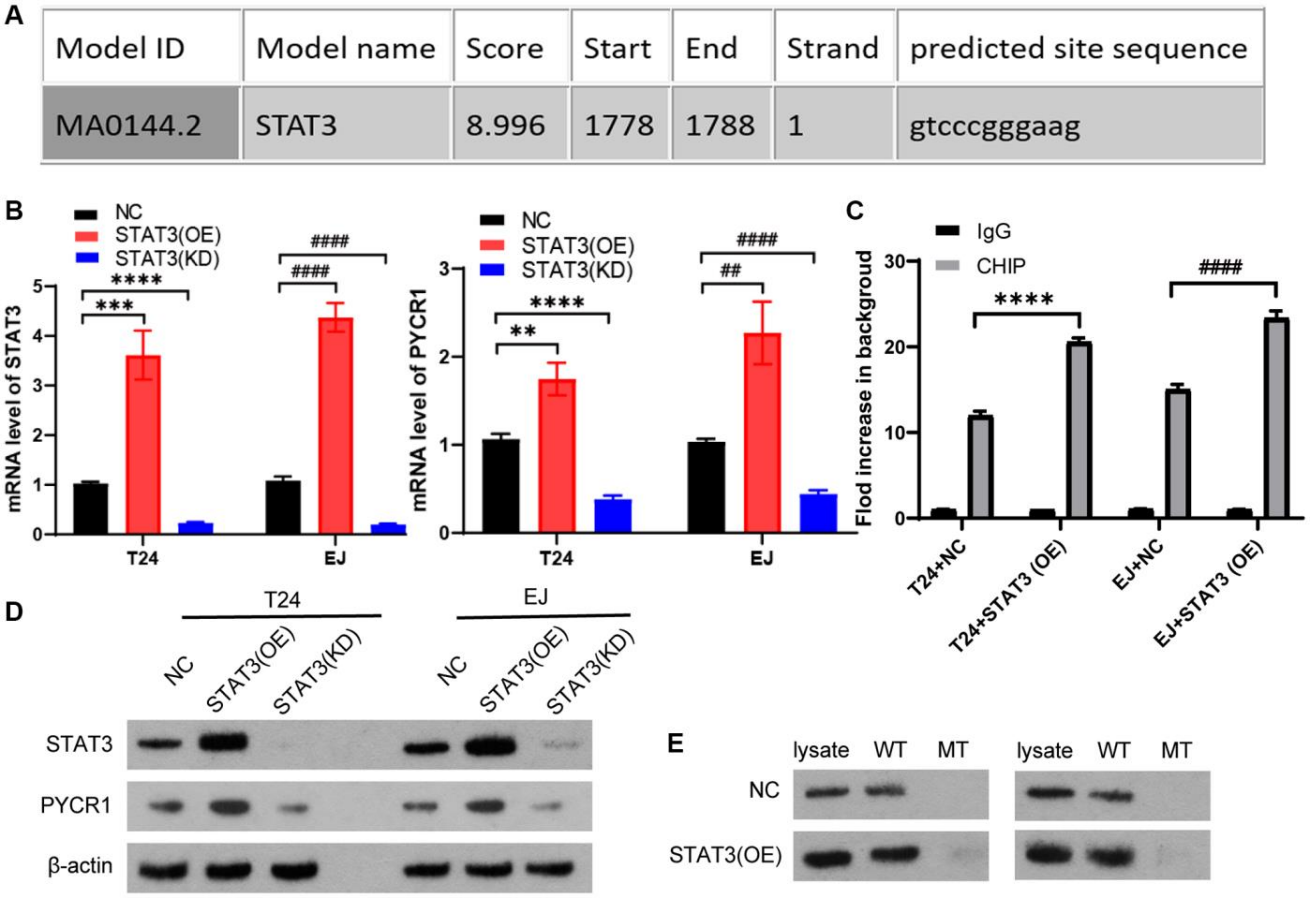
**STAT3 promotes gene and protein levels of PYCR1 by binding to promoter of PYCR1.** (**A**) We predicted that STAT3 could potentially bind to the promoter region of PYCR1 gene by using the jaspar transcription interaction website. T24 and EJ cells were transfected plasmids with overexpression of STAT3 [STAT3(OE)] and knock-down of STAT3[STAT3(KD)]. (**B**) The mRNA level of SENP3 and STAT3 measured by qPCR in NC, STAT3(OE), STAT3(KD) T24 and EJ cells. All data in this figure are represented as mean ± SD. ^*^P < 0.05, compared in T24 cells; ^#^*P* < 0.05, compared in EJ cells. (**C**) The regulation of STAT3 on promoter region of ZNF667, determined by ChIP assay. [mean ± S.D. (error bars), *n* = 4. ^****^p ≤ 0.0001, compared in T24 cells; ^####^*p* ≤ 0.0001, compared in EJ cells, two-way analysis of variance] (**D**) STAT3 and PYCR1 protein level of NC, STAT3(OE), STAT3(KD) T24 and EJ cells. (**E**) The high score region of the predicted binding sites between PYCR1 promoter and STAT3 protein by DNA-affinity precipitation assay (DAPA), the oligonucleotide DNA probe containing the above binding region and the corresponding mutation probe were designed for DAPA detection. All experiments were performed in triplicates.

Our team have found that PYCR1 is highly expressed in bladder cancer and has an oncogene function [26]. In this study, we want to know the upstream regulator of PYCR1. We predicted that STAT3 could potentially bind to the promoter region of PYCR1 gene by using the jaspar transcription interaction website. Therefore, it can be assumed that STAT3 as a transcription factor has the function of activating PYCR1 in bladder cancer (Figure 4A). To investigate the specific regulatory mechanism of STAT3 on PYCR1, we constructed STAT3 stably overexpression and knockdown cells. STAT3 promotes the gene and protein expression levels of PYCR1 (Figure 4B and 4D). We predicted that STAT3 could potentially bind to the PyCR1 promoter region by using the Jaspar transcriptional interaction website, and the results of immunoprecipitation experiment showed that overexpression of STAT3 could increase the enrichment of STAT3 protein to the PyCR1 promoter region (Figure 4C). DNA-affinity Precipitation Assay (DAPA) results show that PYCR1 promoter binds to the STAT3 protein (Figure 4E). These results indicate that STAT3 promotes gene and protein levels of PYCR1 by binding to promoter of PYCR1.

### PYCR1 mitigates the carcinogenic effect of STAT3

**Figure 5:**
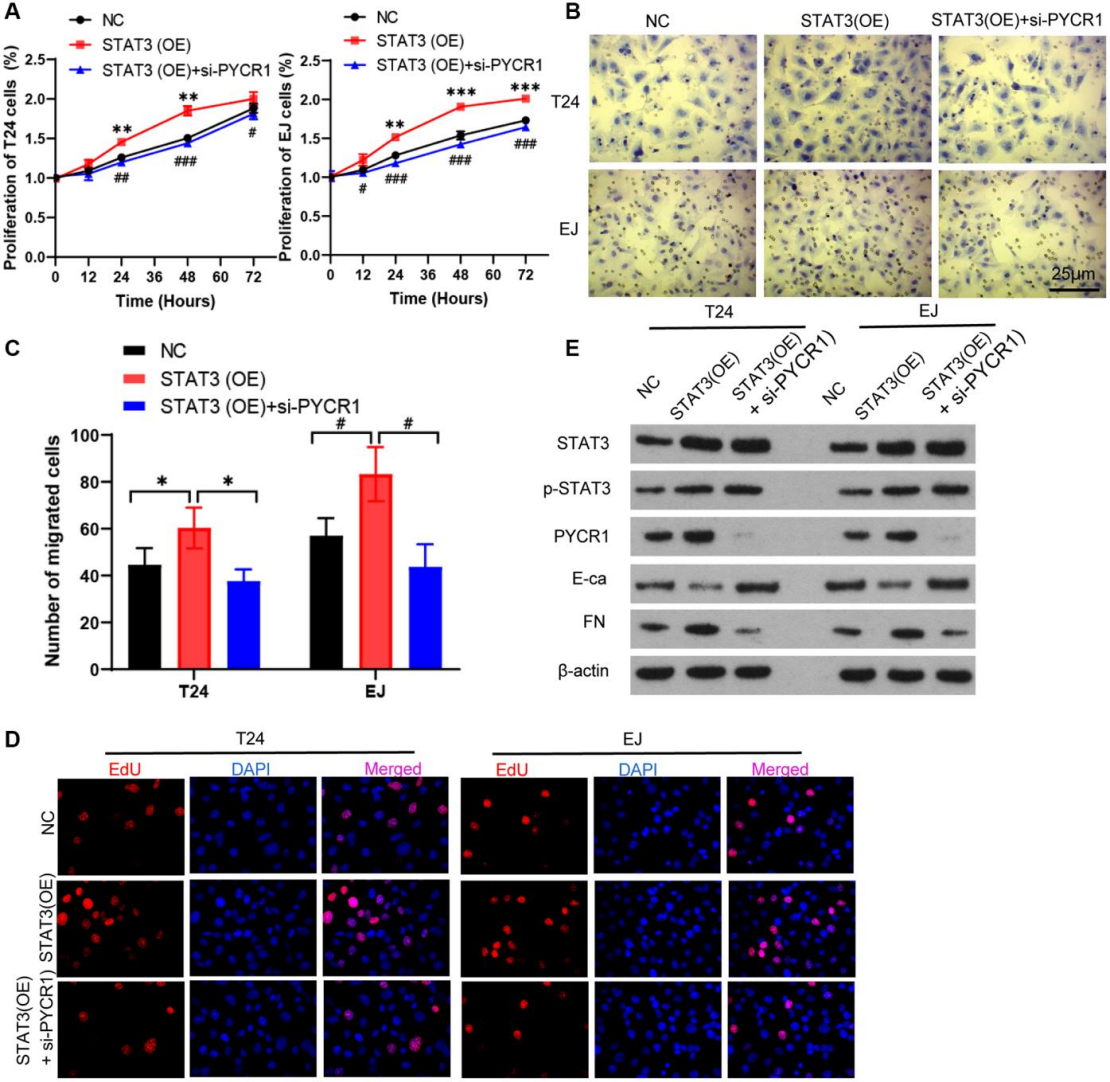
**PYCR1 mitigates the carcinogenic effect of STAT3.** (**A**) Overexpression of STAT3 [STAT3(OE)] T24 and EJ cells were transfected with PYCR1 siRNAs [STAT3(OE) + si-PYCR1]. Cell proliferation were determined by CCK8 essay in NC, SENP3(OE), SENP3(OE) + si-STAT3 T24 and EJ cells. [mean ± S.D. (error bars), *n* = 3. ^**^p ≤ 0.01; ^***^p ≤ 0.001, compared with NC group; ^#^*p* ≤ 0.05; ^##^*p* ≤ 0.01; ^###^*p* ≤ 0.001, compared with STAT3(OE) group, two-way analysis of variance]. (**B**) Cell invasion determined by trans well staining in NC, STAT3(OE), STAT3(OE) + si-PYCR1 T24 and EJ cells. (**C**) Analysis of cell migration. All data in this figure are represented as mean ± SD. ^*^P < 0.05, compared in T24 cells; ^#^*P* < 0.05, compared in EJ cells. (**D**) EDU positive cells in in NC, SENP3(OE), SENP3(OE) + si-STAT3 T24 and EJ cells determined by Confocal immunofluorescence. (**E**) Abundance of the indicated protein was analyzed by Western blotting. All experiments were performed in triplicates.

To test whether knock-down of PYCR1 reduces the carcinogenic effect of STAT3, we transfected with PYCR1 siRNA in overexpression of STAT3 cells. Compared with normal cells, overexpression of STAT3 promoted cell proliferation, invasion and EDU proliferation. However, silencing PYCR1 in overexpression of STAT3 cells completely rescued the cell proliferation and invasion to normal cells (Figure 5A–5D). Simultaneously, silencing PYCR1 significantly up-regulated p-STAT3 protein level (Figure 5E). STAT3 reduced E-Ca and increased FN protein levels. When knocking down PYCR1 of overexpression of STAT3 cells, the changes of E-Ca and FN could be reversed (Figure 5E). Together, PYCR1 mitigates the carcinogenic effect of STAT3.

### SENP3 promotes tumor proliferation by upregulating STAT3 *in vivo*

**Figure 6:**
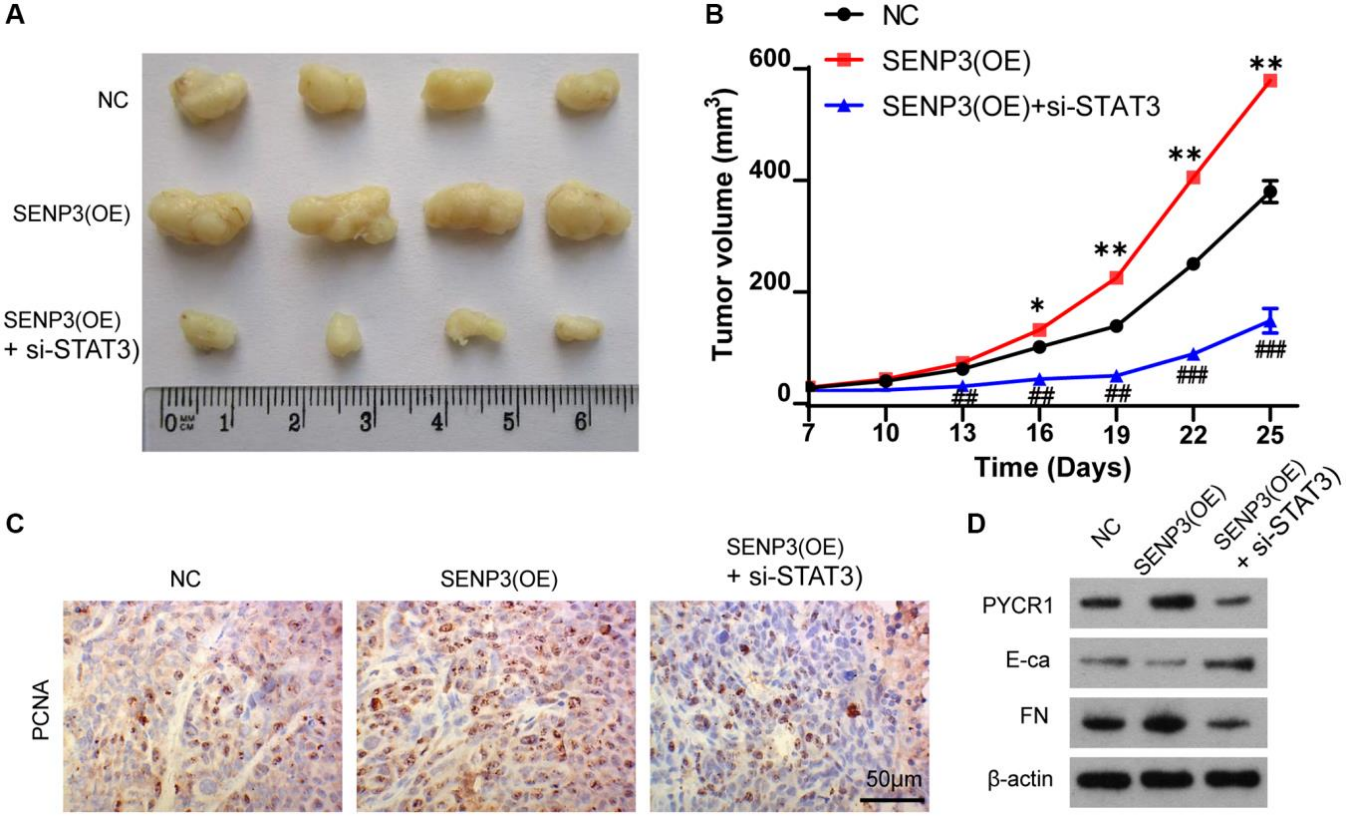
**SENP3 promotes tumor proliferation by upregulating STAT3 *in vivo*.** (**A**) A subcutaneous transplantation tumor model in nude BALB/c mice was established using the human bladder cancer cell line T24. The mice were divided into NC, SENP3(OE) and SENP3(OE) + si-STAT3 groups with 5 mice per group. (**B**) The growth curve of tumor volumes. [mean ± S.D. (error bars), *n* = 4. ^*^p ≤ 0.05; ^**^p ≤ 0.01, compared with NC group; ^##^*p* ≤ 0.01; ^###^*p* ≤ 0.001, compared with STAT3(OE) group, two-way analysis of variance]. (**C**) PCNA positive cells in subcutaneous transplantation tumor isolates by immunohistochemistry. [scale bar, 50 μm]. (**D**) PYCR1, E-ca and FN protein level of NC, SENP3(OE) and SENP3(OE) + si-STAT3 mice transplantation tumor.

The nude BALB/c mice with bladder cancer T24 cell were used for the study *in vivo*. The mice were divided into NC, SENP3(OE) and SENP3(OE) + si-STAT3 groups with 5 mice per group. Overexpression of SENP3 accelerated tumor growth compared with control group (Figure 6A and 6B). However, knocking down STAT3 reduced tumor growth (Figure 6A and 6B). The PCNA staining showed that knocking down STAT3 inhibited cell proliferation promoted by SENP3 (Figure 6C). SENP3 upregulated PYCR1 protein level and promoted EMT transformation, but silencing STAT3 reversed the effect of SENP3 (Figure 6D). Taken together, SENP3 promotes bladder cancer proliferation and EMT transformation by regulating STAT3.

## DISCUSSION

BC currently afflicts 430,000 patients and causes 165,000 deaths per year worldwide [29–31]. To enhance bladder cancer therapy, new molecular targets for its diagnosis and prognosis must be identified, and new treatments must be developed. This study found that SENP3 induced deSUMOylation of STAT3 remarkably promote bladder cancer proliferation and EMT. It is known that STAT3 transcriptional activity is mainly regulated protein PTMs [32–37]. Among the PTMs, SUMOylation is critical for STAT3 transcriptional activity [38, 39]. SUMOylation of STAT3 negatively regulates its activity by restraining Y705 phosphorylation in the nucleus in head and neck cancer [16]. Previous reports have showed that SENP3 regulates deSUMOylation of chromosome-associated proteins, influencing many biological processes [36, 40]. However, SENP3 has been found to be highly expressed in malignant tumors, such as head and neck cancer [16], ovarian cancer [41], and gastric cancer [40]. Here, we found that SENP3 expression was higher in bladder cancer tissues than in normal tissues. Moreover, SENP3 promoted the proliferation of bladder cancer cells. SENP3 elevated STAT3 protein level, but did not influence the gene expression, suggesting that SENP3 regulated STAT3 via PTM approach. Further study showed that SENP3 enhanced STAT3 protein stability by facilitating STAT3-deSUMOylation. As the results, SENP3 significantly promoted the p-STAT3 protein level, and induced the distribution of p-STAT3 in the nucleus. Knock-down of STAT3 impaired the carcinogenic effect of SENP3. These results indicate that the carcinogenic effect of SENP3 is associated to the increase of STAT3 protein levels.

PYCR family proteins are implicated in proline biosynthesis and other cell metabolism [42, 43]. Recent study has found that PYCR1 is highly expressed in bladder cancer; depletion of PYCR1 in turn suppresses proliferation and invasion of bladder cancer [44]. This result indicates that abnormal up-regulated PYCR1 participates in the development of bladder cancer. The Jaspar transcriptional interaction website predicted that STAT3 could potentially bind to the *PYCR1* gene promoter region. In this study, we confirmed that STAT3 interacts with the promoter of *PYCR1* gene and is responsible for the PYCR1 transcription. Through this way, STAT3 promoted PYCR1 gene expression, resulting in the increased PYCR1 protein. Knock-down of PYCR1 inhibited the viability, proliferation, invasion and EMT of bladder cancer cells that were induced by STAT3. These data indicate that the carcinogenic effects of STAT3 are relied on inducing PYCR1 expression.

There is a limitation in this study. We found that SENP3 enhanced STAT3 protein stability by facilitating STAT3-deSUMOylation, but it is still unclear why deSUMOylation of STAT3 influences STAT3 protein stability. It is known that there is close interaction among various PTMs, such as the interaction between SUMOylation and ubiquitination, the interaction between SUMOylation and phosphorylation, as well as the interaction between phosphorylation and ubiquitination. It is possible that deSUMOylation of STAT3 impacts the ubiquitination, whereby influencing STAT3 protein stability. Further study was warranted to identify the hypothesis.

**Figure 7:**
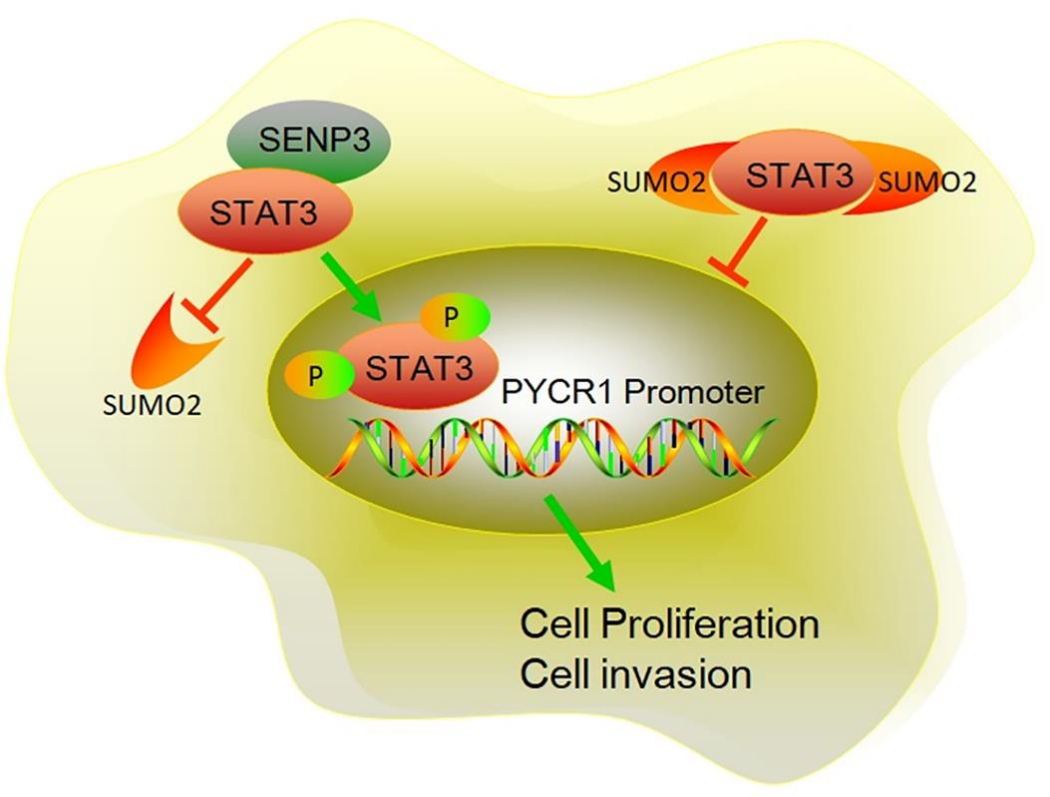
**The mechanism diagram of SENP3 promoting BC proliferation and invasion by upregulating STAT3.** SENP3 induces deSUMOylation of STAT3, which promotes the phosphorylation of STAT3. Phosphorylation of STAT3 induces STAT3 translocating into nuclear. As a transcriptional factor, nuclear STAT3 promotes PYCR1 expression, resulting enhanced proliferation and invasion of BC.

In all, this study tested STAT3 and SENP3 expression in bladder cancer tissues and examined its connection and molecular mechanism in bladder cancer. We found that SENP3 enhanced STAT3 protein stability by facilitating STAT3-deSUMOylation. As the result, both the total and phosphorylated STAT3 were increased by SENP3. Phosphorylated STAT3 translocated to nuclear and then initiate the transcription of *PYCR1* gene. SENP3/STAT3/PYCR1 pathway promoted the viability, proliferation, invasion and EMT of bladder cancer cells (Figure 7). The results provide novel insights into the regulation of SENP3/STAT3/PYCR1 pathway, which has key roles in bladder cancer progression and may aid in identifying new biomarkers or targeted therapies for bladder cancer.

## MATERIALS AND METHODS

### Immunohistochemistry (IHC)

Tissue samples were obtained from Hunan Provincial People’s Hospital. These samples were deparaffinized at 60°C for half an hour and then permeabilized with 0.5% Triton X-100 in PBS for 20 min at room temperature. Blocking buffer (0.5% BSA + 5% goat serum) was used to block for 30 min at room temperature. Primary antibody was diluted in blocking buffer (1:100 or 1: 200), and added to the sample with the incubation overnight at 4°C. Secondary Antibody (Biotin-labeled secondary antibody, 1:200, diluted in blocking buffer) was added with incubation for 30 min at room temperature. ABC dilution (1 ml PBS + 10 ul A + 10 ul B) were configured half an hour. DAB Chromogenic solution was used for visualization.

### Cell culture and transfection

Bladder cancer cell lines, T24 and EJ, were cultured in the dulbecco’s modified eagle medium (DMEM) with 10% serum. The cells were proliferated to 80% density for passage. The preparation of the overexpressed plasmid-Lipofectamine™ 3000 complex was as follows: 7.5 μl Lipofectamine™ 3000 was diluted with 125 μl Opti-Mem^®^ I, and 5 μl plasmid diluted with 125 μl Opti-Mem^®^ I. P3000TM reagent (5 μl) was then mixed with the diluted plasmid and Lipofectamine™ 3000 and incubated at room temperature for 5 min. Cells (5 × 10^4^) were seeded on 6-well plates to achieve 70–90% cell convergence within 24 hours. The plasmid-Lipofectaminet™ 3000 complex was added to each well, followed by the incubation for 3–5 hours for downstream experiments.

### Fluorescence staining

Cells in cell slides were fixed with 4% paraformaldehyde for 15 min, permeabilized with 0.5% TritonX-100 for 20 min and blocked for 30 min at room temperature. Cells were further incubated with primary antibody at 4°C overnight, followed by anti-rabbit secondary antibody conjugated with Alexa Flour 488 at room temperature for 1 h and DAPI staining.

### Quantitative real-time PCR (RT-PCR)

The cDNA was prepared, followed by the amplification of the target gene. The RT-PCR reaction solution were prepared by mixing 2× Master mix 10.0 (μl), Forward Primer 1.0 (μl), Reverse Primer 1.0 (μl), nuclease-free H_2_O 1.0 (μl), Total per Reaction 18.0 (μl). The difference of gene expression was detected by fluorescence quantitative PCR.

### Western blot assay

Tissues and cells were collected and resuspended with an appropriate volume of RIPA lysate for 30 min on ice. The different treated samples with 30 μg total protein were separated on SDS-PAGE and transferred to PVDF membrane. Protein expressions were blocked with 5% skim milk for 1 h at room temperature. Primary antibody, diluted in the blocking solution, was incubate overnight at 4°C; and the secondary antibody was for 1 h at room temperature. Finally, the membranes were visualized using Pierce^®^ ECL Western Blotting. Primary antibody: STAT3 (Abcam, ab32500), p-STAT3 (Abcam, ab76315), FN (promab, 30506), E-cad (Ptgcn, 20874-1-AP), SENP3 (Ptgcn, 17659-1-AP), Sumo 2 (Abcam, ab233222), PYCR1 (Ptgcn, 66510-1-Ig), β-actin (Ptgcn, 66009-1-Ig).

### Transwell

Cells were treated with drugs (or not) for a certain time. Add the serum culture medium containing 20% FBS into wells (600 μl/well). Put trans well chambers in 24-well plates. Digest cells and resuspend cells with serum-free culture medium. Cell concentration should be determined according to specific conditions. Add cell suspension into trans well chambers (400 μl/chamber). Put it in an incubator for a certain period of time. Determine the time by checking the literature. Take trans well chambers out and wipe cells that have not passed through the inside of the microporous filter with a cotton swab. Fix cells methanol with for 5 minutes. Dye cells with purple crystal and clean trans well chambers with pure water. Under the microscope, take five fields (top, bottom, left, right, middle) and take pictures.

### Extraction of plasma/nucleoprotein

NucBuster TM Protein Exaction Kit (Merck Millipore, 71183-3) was used to extract plasma/nucleoprotein as the flowing method. The collected cells were added with liquid nitrogen and quickly ground into a powder. Appropriate amount of powder was diluted with 150 ul NucBuster Reagent 1, then vortexed with high speed for 15 S. The samples were placed on ice for 5 min and centrifuged with 16000 g at 4°C for 20 min. The supernatant was served as cytoplasmic protein, and floccule was added 1 ul 100 × Protease Inhibitor Cocktail, 1 ul 100 mM DTT and 75 ul NucBuster Exaction Reagent. The mixture was vortexed with high speed for 15 S and placed on ice for 5 min. Then the mixture centrifuged with 16000 g at 4°C for 20 min. Cytoplasmic protein and nucleoprotein can be used immediately or stored in separate packages at −80°C.

### CCK8 assay

Cells in a 96-well plate were treated with agents for 1 h. Finally, the absorbance was read at 550 nm using a microplate reader (Thermo Fisher Scientific, Franklin, MA, USA). Cell growth was calculated using formula: (OD value of test well - OD value of background control well)/(OD value of control cell - OD value of background control) × 100%.

### CHIP

The cells were fixed in 1% formaldehyde and incubated at room temperature for 10 minutes. 1 mL of 10× Glycine was added to each dish to quench unreacted formaldehyde and incubate at room temperature for 5 minutes. These dishes were placed on ice. Medium was removed as much as possible. Each dish was added 2 mL cold PBS containing Protease Inhibitor Cocktail. The cells were collected with 800 g at 4ºC for 5 minutes. During spin, each sample was combined with 0.5 mL of cell Lysis Buffer with 2.5 μL of Protease Inhibitor Cocktail II.

### Tumor formation assay in nude mice

Male BALB/c nude mice (6-week-old) were maintained in pathogen-free conditions. The mice were injected subcutaneously into the right flanks with 1 × 10^6^ cells/mL (0.1 mL) of T24 cells, which were stably transfected with pLentiCon/NC, plentiSENP3/OE, plentiSENP3 + si-STAT3/OE + si-STAT3 plasmid and siRNA. Tumor growth was examined every 7 days, and tumor volumes were calculated using the equation, V = 0.52 × length × width^2^. All animal care and experiments were conducted in accordance with national and institutional policies for animal health and well-being. The protocol was approved by the Institutional Animal Care and Use Committee.

### Statistical analysis

All statistical tests were performed using an unpaired two-tailed Student’s *t* test for two data sets when the data met the normal distribution tested by F-test or one-way ANOVA followed by the Scheffe’s post-hoc test for multiple comparisons. *p* < 0.05 was considered to a statistically significant difference.

### Data availability

The data used to support the findings of this study are available from the corresponding author upon request.

## References

[1] Gupta B, Kumar N. Worldwide incidence, mortality and time trends for cancer of the oesophagus. Eur J Cancer Prev. 2017; 26:107–18. 10.1097/CEJ.0000000000000249 27014938

[2] Hung CF, Yang CK, Ou YC. Urologic cancer in Taiwan. Jpn J Clin Oncol. 2016; 46:605–9. 10.1093/jjco/hyw038 27052114

[3] Wong MCS, Fung FDH, Leung C, Cheung WWL, Goggins WB, Ng CF. The global epidemiology of bladder cancer: a joinpoint regression analysis of its incidence and mortality trends and projection. Sci Rep. 2018; 8:1129. 10.1038/s41598-018-19199-z 29348548 PMC5773684

[4] Chavan S, Bray F, Lortet-Tieulent J, Goodman M, Jemal A. International variations in bladder cancer incidence and mortality. Eur Urol. 2014; 66:59–73. 10.1016/j.eururo.2013.10.001 24451595

[5] Lapitan MCM, Cody JD, Mashayekhi A. Open retropubic colposuspension for urinary incontinence in women. Cochrane Database Syst Rev. 2017; 7:CD002912. 10.1002/14651858.CD002912.pub7 28741303 PMC6483458

[6] Guo CC, Bondaruk J, Yao H, Wang Z, Zhang L, Lee S, Lee JG, Cogdell D, Zhang M, Yang G, Dadhania V, Choi W, Wei P, et al. Assessment of Luminal and Basal Phenotypes in Bladder Cancer. Sci Rep. 2020; 10:9743. 10.1038/s41598-020-66747-7 32546765 PMC7298008

[7] Ng K, Stenzl A, Sharma A, Vasdev N. Urinary biomarkers in bladder cancer: A review of the current landscape and future directions. Urol Oncol. 2021; 39:41–51. 10.1016/j.urolonc.2020.08.016 32919875

[8] Ma S, Zhu L, Fan X, Luo T, Liu D, Liang Z, Hu X, Shi T, Tan W, Wang Z. Melatonin derivatives combat with inflammation-related cancer by targeting the Main Culprit STAT3. Eur J Med Chem. 2021; 211:113027. 10.1016/j.ejmech.2020.113027 33248852

[9] Liu Y, Liao S, Bennett S, Tang H, Song D, Wood D, Zhan X, Xu J. STAT3 and its targeting inhibitors in osteosarcoma. Cell Prolif. 2021; 54:e12974. 10.1111/cpr.12974 33382511 PMC7848963

[10] Peng W, Dong N, Wu S, Gui D, Ye Z, Wu H, Zhong X. miR-4500 suppresses cell proliferation and migration in bladder cancer via inhibition of STAT3/CCR7 pathway. J Cell Biochem. 2019. [Epub ahead of print]. 10.1002/jcb.29558 31788846

[11] Lai SC, Su YT, Chi CC, Kuo YC, Lee KF, Wu YC, Lan PC, Yang MH, Chang TS, Huang YH. DNMT3b/OCT4 expression confers sorafenib resistance and poor prognosis of hepatocellular carcinoma through IL-6/STAT3 regulation. J Exp Clin Cancer Res. 2019; 38:474. 10.1186/s13046-019-1442-2 31771617 PMC6878666

[12] Cocchiola R, Rubini E, Altieri F, Chichiarelli S, Paglia G, Romaniello D, Carissimi S, Giorgi A, Giamogante F, Macone A, Perugia G, Gurtner A, Eufemi M. STAT3 Post-Translational Modifications Drive Cellular Signaling Pathways in Prostate Cancer Cells. Int J Mol Sci. 2019; 20:1815. 10.3390/ijms20081815 31013746 PMC6514970

[13] Huang G, Yan H, Ye S, Tong C, Ying QL. STAT3 phosphorylation at tyrosine 705 and serine 727 differentially regulates mouse ESC fates. Stem Cells. 2014; 32:1149–60. 10.1002/stem.1609 24302476 PMC4181708

[14] Lau WW, Ng JK, Lee MM, Chan AS, Wong YH. Interleukin-6 autocrine signaling mediates melatonin MT(1/2) receptor-induced STAT3 Tyr(705) phosphorylation. J Pineal Res. 2012; 52:477–89. 10.1111/j.1600-079X.2011.00965.x 21954831

[15] Ge Q, Lu M, Ju L, Qian K, Wang G, Wu CL, Liu X, Xiao Y, Wang X. miR-4324-RACGAP1-STAT3-ESR1 feedback loop inhibits proliferation and metastasis of bladder cancer. Int J Cancer. 2019; 144:3043–55. 10.1002/ijc.32036 30511377

[16] Zhou Z, Wang M, Li J, Xiao M, Chin YE, Cheng J, Yeh ET, Yang J, Yi J. SUMOylation and SENP3 regulate STAT3 activation in head and neck cancer. Oncogene. 2016; 35:5826–38. 10.1038/onc.2016.124 27181202 PMC5116054

[17] Zhang Y, Zheng LM, Wang CX, Gu JM, Xue S. SENP3 protects H9C2 cells from apoptosis triggered by H/R via STAT3 pathway. Eur Rev Med Pharmacol Sci. 2018; 22:2778–86. 10.26355/eurrev_201805_14975 29771430

[18] Hindupur SV, Schmid SC, Koch JA, Youssef A, Baur EM, Wang D, Horn T, Slotta-Huspenina J, Gschwend JE, Holm PS, Nawroth R. STAT3/5 Inhibitors Suppress Proliferation in Bladder Cancer and Enhance Oncolytic Adenovirus Therapy. Int J Mol Sci. 2020; 21:1106. 10.3390/ijms21031106 32046095 PMC7043223

[19] Chang HM, Yeh ETH. SUMO: From Bench to Bedside. Physiol Rev. 2020; 100:1599–619. 10.1152/physrev.00025.2019 32666886 PMC7717128

[20] Kunz K, Piller T, Müller S. SUMO-specific proteases and isopeptidases of the SENP family at a glance. J Cell Sci. 2018; 131:jcs211904. 10.1242/jcs.211904 29559551

[21] Tokarz P, Woźniak K. SENP Proteases as Potential Targets for Cancer Therapy. Cancers (Basel). 2021; 13:2059. 10.3390/cancers13092059 33923236 PMC8123143

[22] Bhagwat NR, Owens SN, Ito M, Boinapalli JV, Poa P, Ditzel A, Kopparapu S, Mahalawat M, Davies OR, Collins SR, Johnson JR, Krogan NJ, Hunter N. SUMO is a pervasive regulator of meiosis. Elife. 2021; 10:e57720. 10.7554/eLife.57720 33502312 PMC7924959

[23] Li YJ, Du L, Wang J, Vega R, Lee TD, Miao Y, Aldana-Masangkay G, Samuels ER, Li B, Ouyang SX, Colayco SA, Bobkova EV, Divlianska DB, et al. Allosteric Inhibition of Ubiquitin-like Modifications by a Class of Inhibitor of SUMO-Activating Enzyme. Cell Chem Biol. 2019; 26:278–88.e6. 10.1016/j.chembiol.2018.10.026 30581133 PMC6524651

[24] Long X, Zhao B, Lu W, Chen X, Yang X, Huang J, Zhang Y, An S, Qin Y, Xing Z, Shen Y, Wu H, Qi Y. The Critical Roles of the SUMO-Specific Protease SENP3 in Human Diseases and Clinical Implications. Front Physiol. 2020; 11:558220. 10.3389/fphys.2020.558220 33192553 PMC7662461

[25] Weijin F, Zhibin X, Shengfeng Z, Xiaoli Y, Qijian D, Jiayi L, Qiumei L, Yilong C, Hua M, Deyun L, Jiwen C. The clinical significance of PYCR1 expression in renal cell carcinoma. Medicine (Baltimore). 2019; 98:e16384. 10.1097/MD.0000000000016384 31305441 PMC6641676

[26] Song W, Yang K, Luo J, Gao Z, Gao Y. Dysregulation of USP18/FTO/PYCR1 signaling network promotes bladder cancer development and progression. Aging (Albany NY). 2021; 13:3909–25. 10.18632/aging.202359 33461172 PMC7906198

[27] Christensen EM, Bogner AN, Vandekeere A, Tam GS, Patel SM, Becker DF, Fendt SM, Tanner JJ. *In crystallo* screening for proline analog inhibitors of the proline cycle enzyme PYCR1. J Biol Chem. 2020; 295:18316–27. 10.1074/jbc.RA120.016106 33109600 PMC7939384

[28] Wang D, Wang L, Zhang Y, Yan Z, Liu L, Chen G. PYCR1 promotes the progression of non-small-cell lung cancer under the negative regulation of miR-488. Biomed Pharmacother. 2019; 111:588–95. 10.1016/j.biopha.2018.12.089 30605882

[29] Antoni S, Ferlay J, Soerjomataram I, Znaor A, Jemal A, Bray F. Bladder Cancer Incidence and Mortality: A Global Overview and Recent Trends. Eur Urol. 2017; 71:96–108. 10.1016/j.eururo.2016.06.010 27370177

[30] Bray F, Ferlay J, Soerjomataram I, Siegel RL, Torre LA, Jemal A. Global cancer statistics 2018: GLOBOCAN estimates of incidence and mortality worldwide for 36 cancers in 185 countries. CA Cancer J Clin. 2018; 68:394–424. 10.3322/caac.21492 30207593

[31] Nakayama M, Ito Y, Hatano K, Nakai Y, Kakimoto KI, Miyashiro I, Nishimura K. Impact of sex difference on survival of bladder cancer: A population-based registry data in Japan. Int J Urol. 2019; 26:649–54. 10.1111/iju.13955 30916420

[32] Wen Z, Zhong Z, Darnell JE Jr. Maximal activation of transcription by Stat1 and Stat3 requires both tyrosine and serine phosphorylation. Cell. 1995; 82:241–50. 10.1016/0092-8674(95)90311-9 7543024

[33] Busch S, Renaud SJ, Schleussner E, Graham CH, Markert UR. mTOR mediates human trophoblast invasion through regulation of matrix-remodeling enzymes and is associated with serine phosphorylation of STAT3. Exp Cell Res. 2009; 315:1724–33. 10.1016/j.yexcr.2009.01.026 19331815

[34] Yang J, Huang J, Dasgupta M, Sears N, Miyagi M, Wang B, Chance MR, Chen X, Du Y, Wang Y, An L, Wang Q, Lu T, et al. Reversible methylation of promoter-bound STAT3 by histone-modifying enzymes. Proc Natl Acad Sci U S A. 2010; 107:21499–504. 10.1073/pnas.1016147107 21098664 PMC3003019

[35] Zhong Z, Wen Z, Darnell JE Jr. Stat3: a STAT family member activated by tyrosine phosphorylation in response to epidermal growth factor and interleukin-6. Science. 1994; 264:95–8. 10.1126/science.8140422 8140422

[36] Jiang X, Jia X, Sun J, Qi C, Lu L, Wang Y, Zhang L, Wei M. Overexpressed coiled-coil domain containing protein 8 (CCDC8) mediates newly synthesized HIV-1 Gag lysosomal degradation. Sci Rep. 2020; 10:11416. 10.1038/s41598-020-68341-3 32651437 PMC7351720

[37] Ulane CM, Rodriguez JJ, Parisien JP, Horvath CM. STAT3 ubiquitylation and degradation by mumps virus suppress cytokine and oncogene signaling. J Virol. 2003; 77:6385–93. 10.1128/jvi.77.11.6385-6393.2003 12743296 PMC155014

[38] Yeh ET, Gong L, Kamitani T. Ubiquitin-like proteins: new wines in new bottles. Gene. 2000; 248:1–14. 10.1016/s0378-1119(00)00139-6 10806345

[39] Rosonina E, Akhter A, Dou Y, Babu J, Sri Theivakadadcham VS. Regulation of transcription factors by sumoylation. Transcription. 2017; 8:220–31. 10.1080/21541264.2017.1311829 28379052 PMC5574528

[40] Lao Y, Yang K, Wang Z, Sun X, Zou Q, Yu X, Cheng J, Tong X, Yeh ETH, Yang J, Yi J. DeSUMOylation of MKK7 kinase by the SUMO2/3 protease SENP3 potentiates lipopolysaccharide-induced inflammatory signaling in macrophages. J Biol Chem. 2018; 293:3965–80. 10.1074/jbc.M117.816769 29352108 PMC5857993

[41] Cheng J, Su M, Jin Y, Xi Q, Deng Y, Chen J, Wang W, Chen Y, Chen L, Shi N, Mao G. Upregulation of SENP3/SMT3IP1 promotes epithelial ovarian cancer progression and forecasts poor prognosis. Tumour Biol. 2017; 39:1–12. 10.1177/1010428317694543 28351334

[42] Chen S, Yang X, Yu M, Wang Z, Liu B, Liu M, Liu L, Ren M, Qi H, Zou J, Vucenik I, Zhu WG, Luo J. SIRT3 regulates cancer cell proliferation through deacetylation of PYCR1 in proline metabolism. Neoplasia. 2019; 21:665–75. 10.1016/j.neo.2019.04.008 31108370 PMC6526305

[43] Kuo ML, Lee MB, Tang M, den Besten W, Hu S, Sweredoski MJ, Hess S, Chou CM, Changou CA, Su M, Jia W, Su L, Yen Y. PYCR1 and PYCR2 Interact and Collaborate with RRM2B to Protect Cells from Overt Oxidative Stress. Sci Rep. 2016; 6:18846. 10.1038/srep18846 26733354 PMC4702135

[44] Du S, Sui Y, Ren W, Zhou J, Du C. PYCR1 promotes bladder cancer by affecting the Akt/Wnt/β-catenin signaling. J Bioenerg Biomembr. 2021; 53:247–58. 10.1007/s10863-021-09887-3 33689096

